# Complete Genome Sequence of *Streptomyces clavuligerus* F613-1, an Industrial Producer of Clavulanic Acid

**DOI:** 10.1128/genomeA.01020-16

**Published:** 2016-09-22

**Authors:** Guangxiang Cao, Chuanqing Zhong, Gongli Zong, Jiafang Fu, Zhong Liu, Guimin Zhang, Ronghuo Qin

**Affiliations:** a Shandong Medicinal Biotechnology Centre, Shandong Academy of Medical Sciences, Jinan, China; b Shandong Jianzhu University, Jinan, China; c National Engineering Research Center of Chiral Drugs, Lunan Pharmaceutical Group Corporation, Linyi, China

## Abstract

*Streptomyces clavuligerus* strain F613-1 is an industrial strain with high-yield clavulanic acid production. In this study, the complete genome sequence of *S. clavuligerus* strain F613-1 was determined, including one linear chromosome and one linear plasmid, carrying numerous sets of genes involving in the biosynthesis of clavulanic acid.

## GENOME ANNOUNCEMENT

*Streptomyces clavuligerus* is a Gram-positive soil-dwelling bacterium and capable of producing a number of β-lactam metabolites, including the nonclassical β-lactamase inhibitor clavulanic acid (1). Clavulanic acid has been used clinically for the treatment of infectious diseases caused by pathogenic microorganisms resistant to β-lactam antibiotics.

The draft genome sequence of the *S. clavuligerus* type strain ATCC 27064 was reported in 2010, and the genome consists of one linear chromosome and four linear plasmids, named pSCL1, pSCL2, pSCL3, and pSCL4, respectively (2, 3). There are three gene clusters involved in the biosynthesis of clavulanic acid and other clavam metabolites, including the clavulanic acid gene cluster, the clavam gene cluster, and the paralog gene cluster (4, 5). The clavulanic acid gene cluster and the clavam gene cluster are located on the chromosome, approximately 1.4 Mb away. In contrast, both the paralog gene cluster for clavulanic acid and clavam production are located on pSCL4. Genome annotation also revealed that *S. clavuligerus* has the potential to produce dozens of secondary metabolites, including clavulanic acid, cephamycin C, 5S clavams, holomycin, polyketides, and other nonribosomal peptides (2, 6).

A complete genome sequence of an important industrial producer, *S. clavuligerus* F613-1 (7), was reported in this study, which showed good clavulanic acid production and poor ability to produce 5S clavam compounds.

*S. clavuligerus* strain F613-1 was grown in 50 ml of tryptic soy broth medium (Oxoid, United Kingdom) in a 250-ml flask. The flasks were transferred to the rotary shaker (New Brunswick Scientific, USA) and grown at 25°C and 250 rpm for 60 h to obtain mycelium. Genomic DNA was extracted using the genomic DNA purification kit (Promega, USA), and paired-end (PE) 300-bp sequencing libraries were constructed with Ultra DNA library prep kit for Illumina (NEB, United Kingdom). PacBio 10-kb sequencing libraries were constructed with the SMRTbell template prep kit (Pacific Biosciences, USA). The genome sequence was determined using Illumina HiSeq 2500 (Illumina) and PacBio RS II (Pacific Biosciences). *De novo* assembly using SPAdes Genome Assembler 3.8 (Illumina) and Hierarchical Genome Assembly Process 3 (Pacific Biosciences) generated two contigs composed of a chromosome and a plasmid. The chromosomal genome was 6,883,702 bp, with a G+C content of 72.68%. The plasmid was 707,056 bp, with a G+C content of 71.82%. A total of 5,546 genes, including 5,410 coding genes, 18 rRNA, and 65 tRNA were predicted in the chromosomal genome of *S. clavuligerus* strain F613-1. The plasmid was predicted to contain 571 coding genes, and its sequence is nearly identical to, but smaller than, the 1.8-Mb megaplasmid in ATCC 27064 (2).

According to the remarkable features of strain F613-1, this genome sequence can be used to comparatively analyze the genomes of different *S. clavuligerus* wild type and its derivative strains in order to design new strains of *S. clavuligerus* capable of producing higher levels of clavulanic acid.

### Accession number(s).

The complete annotated genome and plasmid sequences of *S. clavuligerus* strain F613-1 were deposited in GenBank under the accession numbers CP016559 and CP016560, respectively.
